# PLGA-based nanoparticles in colorectal cancer immunotherapy: current concepts and future perspectives

**DOI:** 10.3389/fphar.2025.1714460

**Published:** 2025-10-28

**Authors:** Yuhan Wang, Peng Huang, Chun Li, Shengjin Tu, Hua Yang

**Affiliations:** ^1^ Department of General Surgery, Zigong Fourth People’s Hospital, Zigong, Sichuan, China; ^2^ Division of Abdominal Tumor Multimodality Treatment, Cancer Center, West China Hospital, Sichuan University, Chengdu, Sichuan, China

**Keywords:** colorectal cancer, PLGA, nanomedicine, immunotherapy, clinical application

## Abstract

Colorectal cancer (CRC) remains a leading cause of cancer mortality, and the benefits of immune checkpoint inhibitors are largely confined to the dMMR/MSI-H minority, underscoring the need to remodel the immunosuppressive tumor microenvironment (TME). Poly (lactic-co-glycolic acid) (PLGA) nanoparticles offer biodegradable, tunable carriers with high payload capacity and amenability to targeting, enabling precise delivery and controlled release of immunomodulators. In CRC, these platforms can enhance antigen capture and presentation, recondition suppressive myeloid networks, and coordinate checkpoint blockade with complementary therapeutics to strengthen antitumor immunity and restrain tumor growth. In this review, we summarize current principles for PLGA nanoparticles-based immunotherapies, emphasizing payload selection, release kinetics, microenvironmental responsiveness, and spatiotemporal targeting in CRC. We also outline translational considerations encompassing safety, pharmacokinetics, manufacturability, and regulatory readiness. Addressing these factors may accelerate clinical deployment of PLGA-enabled strategies and extend the benefits of immunotherapy in CRC patients.

## 1 Introduction

The GLOBOCAN 2022 has estimated that colorectal cancer (CRC) is the third most commonly diagnosed cancer and the second leading cause of cancer-related deaths (Bray et al., 2024). In 2022, CRC caused over 1,800,000 new cases and nearly 880,000 deaths globally (Bray et al., 2024). Despite advancements in screening and treatment, the incidence and mortality rates of CRC remain high (Filho et al., 2025). Notably, due to the lack of early-diagnosed biomarkers, many patients diagnosed with advanced stages, are not suitable for surgical resection (Lech et al., 2016). These patients will be exposed to systematic treatment regimens, which include chemotherapy, targeted therapy, and radiation therapy (Singh et al., 2024; Li et al., 2024). However, the effectiveness of these existing therapies is limited, hindered by tumor heterogeneity and drug resistance, resulting in a less than 20% 5-year survival rate (Zhang et al., 2024; Ma et al., 2023). These clinical evidence reveals that CRC still poses a heavy burden on global public health.

Immunotherapy modulates the immune response by stimulating the body’s immune defense mechanisms or utilizing bioactive compounds to prevent the onset and progression of tumors (Szeto and Finley, 2019). Over the past decade, immunotherapy has achieved significant advancements in the treatment of various tumors including CRC (Brandenburg et al., 2024; Goodman et al., 2023). Cases that accumulate mutations due to somatic or germline loss of mismatch repair protein function—referred to as microsatellite high (MSI-H) and mismatch repair deficient (dMMR)—exhibit neoantigens on their MHC class I molecules (Pacholczak-Madej et al., 2024; Margalit et al., 2024). This allows for T-cell recognition and activation (Margalit et al., 2024). In contrast, tumors that are microsatellite stable (MSS) or mismatch repair proficient (pMMR) are considered immunologically cold, which have limited efference of immunotherapy (Takei et al., 2024; Williams et al., 2024). Current clinical trials have demonstrated that CRC patients harboring dMMR/MSI-H can benefit from immune checkpoint inhibitors (ICIs) including monoclonal antibodies targeting the programmed cell death receptor-1 (PD-1) and programmed cell death ligand-1 (PD-L1) (Suarez-Carmona and Halama, 2024; Zhu et al., 2023). But the dMMR/MSI-H patients only represent around 15% of all CRC cases (Jin and Sinicrope, 2022). In contrast, ICI therapy is generally ineffective for MSS/pMMR CRC cases, which accounts for the majority of patients (Williams et al., 2024). Moreover, despite the high rate of durable responses, a subset of these patients may develop immunotherapeutic resistance, which can ultimately affect the clinical prognosis (Lyu et al., 2025; Wang et al., 2025). Emerging evidence has indicated that alterations in the tumor microenvironment (TME), play a significant role in the development and progression of CRC, alongside genetic mutations (AlMusawi et al., 2021; Peng et al., 2022). Consequently, the TME in CRC is a critical factor influencing the effectiveness of ICI immunotherapy (Zafari et al., 2022). Therefore, it is essential to explore new strategies that can overcome drug resistance and enhance the efficacy of ICI therapy by modulating the TME.

Nanotechnology has emerged as a rapidly advancing discipline that merges principles from both science and engineering, focusing on the manipulation of materials within the nanoscale, which is typically characterized by sizes ranging from 1 to 1,000 nm (Abaszadeh et al., 2023). Due to their distinctive properties, including high payload capacity, various administration routes, and flexibility for modifications catering to different delivery requirements, there is a growing interest in nanomedicine-based immunotherapy among researchers (Grippin et al., 2025). Notably, poly (lactic-co-glycolic acid) (PLGA) nanoparticles have been identified as prominent nanocarriers owing to their remarkable biocompatibility and biodegradability, rendering them suitable for drug delivery applications (Kesharwani et al., 2025). Their versatility allows for different methods of administration, such as oral or injectable, tailored to meet individual patient preferences (Ahmad et al., 2025). Moreover, surface modifications of PLGA nanoparticles can enhance their targeting abilities and positively affect the TME within CRC (Hu et al., 2025; Silveira et al., 2025). PLGA nanoparticles can foster an immune-activating environment that not only stimulates the activation of effector T cells but also reduces the activity of immunosuppressive cells, such as regulatory T cells (Tregs) and myeloid-derived suppressor cells (MDSCs) (Bahreyni et al., 2025; Yang et al., 2025; Cerioli et al., 2025; Chen et al., 2025a). Furthermore, PLGA nanoparticles can be engineered to co-deliver ICIs alongside conventional anticancer treatments, thus further enhancing the immune response towards cancer (Wei et al., 2025; Wu et al., 2025). This designed targeted delivery mechanism allows for a concentrated effect in tumor tissues, effectively mitigating side effects on healthy cells while optimizing the potency of the drug and improving overall clinical outcomes (Dong et al., 2025; Karatug Kacar et al., 2025). This review will explore the potential of PLGA nanomedicine in modifying the TME of CRC and enhancing anti-tumor immunity. Additionally, the article will highlight the challenges faced by nanotechnology in treating CRC and propose future development directions.

## 2 Current landscape of CRC immunotherapy

Immunotherapy has made significant progress in recent years, but its efficacy for CRC is limited to specific patient groups. ICIs, such as pembrolizumab, nivolumab, and ipilimumab, have been used to treat CRC patients with dMMR or MSI-H (André et al., 2020; Andre et al., 2024; André et al., 2025). Currently, only a small proportion of CRC patients meet the criteria for receiving immunotherapy in clinical practice. In addition to ICIs, various immunotherapy approaches have attracted considerable attention (Dang et al., 2024; Li et al., 2025a). A great number of trials have investigated the potential of these techniques in cancer treatment. Adoptive cell therapy such as chimeric antigen receptor (CAR) T for treating CRC is currently in the preliminary research phase and remains a promising therapeutic avenue for patients who have had poor responses to conventional treatments (Chen et al., 2025b; Chen et al., 2023). However, its early-stage status reflects persistent hurdles including antigenic heterogeneity, the scarcity of CRC-specific targets, an immunosuppressive TME, and impediments to T-cell trafficking, infiltration, and persistence, which significantly impedes clinical translation (Chen et al., 2025b).

Moreover, therapeutic cancer vaccines have demonstrated positive outcomes in terms of safety and feasibility in advanced CRC patients (Jia et al., 2024). For example, phase III trials have shown that combining OncoVax vaccine with chemotherapy have suggested potential benefits as evidenced by advantages in overall survival for CRC patients (Li et al., 2023a; Bastin et al., 2023). These innovative immunotherapies are still underdeveloped in several areas, which contributes to the unsatisfactory efficacy (Albarrán et al., 2024; Baker et al., 2023). Moreover, TME of solid tumors, such as CRC, comprises various cell types that form a complex suppressive network that facilitates tumor growth, and immune escape (Dai et al., 2025; Zhang et al., 2025). These dynamic interactions are essential for the heterogeneity, clonal evolution and drug resistance of malignant cells (Al Zein et al., 2023; Cañellas-Socias et al., 2024). Tumor cells secrete numerous chemokines and cytokines that attract immunosuppressive cells, like MDSCs and regulatory T cells, into the TME (Gallo et al., 2021; Zeng et al., 2024). They also induce the transformation of fibroblasts into cancer-associated fibroblasts (CAFs), which subsequently promote tumor development while inhibiting the function and proliferation of effector T cells (Kamali et al., 2022; Pei et al., 2023). Additionally, transforming growth factor-β (TGFβ) and WNT signaling pathways significantly contribute to immunotherapy resistance in CRC. TGF-β acts across both the innate and adaptive arms of immunity, reshaping the differentiation and effector functions of immune cells. Functionally, TGF-β dampens immune competence by constraining NK-cell development and cytotoxic activity and by impeding the effector maturation of CD8^+^ T cells, collectively blunting antitumor responses (Johansen et al., 2024). Moreover, activation of the WNT–β-catenin axis has been implicated in tumor immune escape. WNT11, a ligand operating within the non-canonical WNT pathway, marks CRC hepatic metastases characterized by exclusion of intratumoral CD8^+^ T lymphocytes and reduced responsiveness to immune checkpoint blockade (Jiang et al., 2025). AdditionallThe composition and characteristics of the TME can be influenced by various immunosuppressive mechanisms that may arise during cancer progression or as a result of therapeutic interventions (Goto et al., 2025; Emami et al., 2025). Thus, a comprehensive understanding of the molecular, cellular, and metabolic features of the TME is essential for elucidating the mechanisms that underlie these processes and for predicting therapeutic outcomes in cancer patients (Padzińsk et al., 2024). Nanoparticles hold significant promise in cancer immunotherapy, as they can be precisely used to deliver immunomodulatory agents directly to the tumor site, thereby altering the local microenvironment (Jiang et al., 2024; Lorenzoni et al., 2025). Compared to conventional therapies, nanoparticle-based approaches can elicit more robust antitumor immune responses with improved specificity and efficacy.

## 3 PLGA-based nanoparticles: new frontiers in cancer immunotherapy

PLGA is a synthetic copolymer that can encapsulate and transport a broad range of cargos, including proteins, peptides and diverse anticancer agents (Su et al., 2021). PLGA is produced via random copolymerization of lactic acid (LA) and glycolic acid (GA), conferring excellent biocompatibility and biodegradability. *In vivo*, the polymer hydrolyzes to LA and GA, which enter the tricarboxylic acid cycle and are ultimately converted to water and carbon dioxide; the carbon dioxide is then eliminated through the lungs (Su et al., 2021). The fabrication of PEGylated PLGA nanoparticles proceeds through several key stages to ensure robust performance (Sun et al., 2024). Selection of the PLGA backbone, specifically its molecular weight and lactic-to-glycolic acid ratio governs hydrolytic degradation and, consequently, drug release profiles (Costa et al., 2025). In parallel, the PEG chain length is chosen to optimize steric stabilization, biocompatibility, and circulation time. A widely adopted approach is the emulsion–solvent evaporation method: the therapeutic payload, PLGA, and PEG are dissolved in a volatile organic phase (e.g., dichloromethane) and dispersed into an aqueous solution containing a stabilizer such as poly (vinyl alcohol) (typically ∼1% w/v) (Shang et al., 2025; Theodosis-Nobelos et al., 2025). High-shear homogenization yields a fine oil-in-water emulsion, after which solvent removal under reduced pressure or prolonged stirring solidifies the particles (Zhang et al., 2023). The resulting nanoparticles are purified by repeated washing with ultrapure water to eliminate residual solvent and surfactant, then characterized by dynamic light scattering for hydrodynamic size and polydispersity and by electrophoretic light scattering (zeta potential) to assess surface charge (Essa et al., 2020; Zhang et al., 2019). The efficacy of immunotherapy is closely tied to efficient delivery to diseased tissues and controlled release of bioactive agents (Rahimian et al., 2015). As a versatile carrier, PLGA can be engineered into nanoparticles that engage the immune system through multiple pathways: enhancing antigen uptake and presentation, modulating inflammatory signaling, amplifying antigen-specific responses, and interfacing directly with key immune cell populations (Horvath and Basler, 2023; Sonam et al., 2023). Owing to its biodegradability, tunable composition, and capacity to co-deliver antigens and adjuvants, PLGA-based nanoplatforms exhibit substantial promise for applications in cancer immunotherapy (Yang et al., 2025; Yang et al., 2021).

PLGA-NPs potentiate antigen processing and presentation, rapidly priming dendritic cells (DCs) and T cells (Tang et al., 2022a). Encapsulation of immunoadjuvants such as CpG oligodeoxynucleotides within PLGA NPs accelerates the activation of natural killer cells and T cells, thereby initiating a brisk immune response (Yang et al., 2018). Kim et al. (Kim et al., 2018) co-delivered an exogenous tumor antigen with an immunostimulant by encapsulating imidazoquinoline ester TLR7/8 agonists into PLGA using an emulsion solvent–evaporation method. TLRs on immune cells induce proinflammatory responses (e.g., IFN-γ) that counter the tolerogenic TME. The resulting PLGA nanoparticles were ∼210 nm with 0.94 µg adjuvant per mg; after subcutaneous administration, they trafficked to draining lymph nodes and co-delivery with soluble OVA significantly reduced OVA-pulsed targets in an *in vivo* cytotoxicity assay. Prophylactically, the vaccine decreased lung metastases in the B16-F10 OVA model and reduced tumor volumes in a B16-F10 melanoma model *versus* soluble antigen/adjuvant and untreated controls (Kim et al., 2018). Encapsulating the double-stranded RNA adjuvant Riboxxim in PLGA nanoparticles robustly primed murine and human DCs and elicited tumor-specific CD8^+^ T-cell responses, outperforming conventional dsRNA analogs. In combination with ICIs, this strategy slowed primary tumor growth, limited metastatic spread, and prolonged survival in mouse models (Koerner et al., 2021). Overall, the tunability of PLGA’s hydrophobic character and degradation kinetics has the potential to be a versatile platform for delivering a broad spectrum of immunotherapeutic cargos.

## 4 PLGA-based nanoparticles in CRC immunotherapy

**FIGURE 1:**
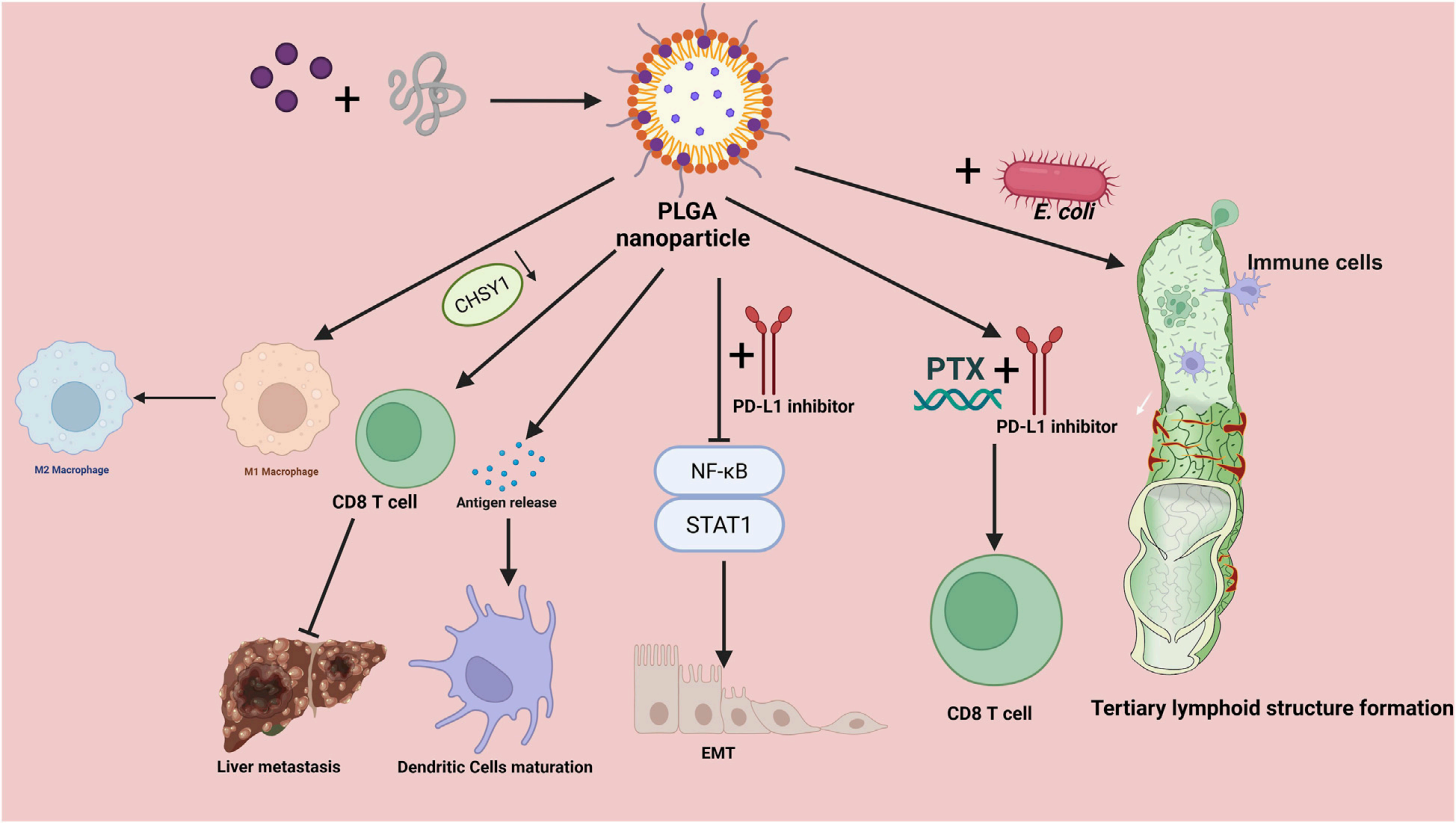
Mechanisms of PLGA-based nanoparticles in colorectal cancer immunotherapy. Drug-loaded poly (lactic-co-glycolic acid) (PLGA) nanoparticles orchestrate multiple antitumor immune processes. Left: NPs promote tumor-antigen release and drive dendritic cell maturation, while reprogramming tumor-associated macrophages from an M2 to an M1 phenotype, thereby enhancing CD8^+^ T-cell priming and cytotoxic activity and ultimately restraining liver metastasis. The CHSY1 axis is highlighted as a regulator linked to CD8^+^ T-cell activation and metastatic propensity. Right: Co-administration with engineered *E. coli* augments innate and adaptive immune activation, supporting the formation of tertiary lymphoid structures within the tumor microenvironment. The schematic also depicts therapeutic combinations in which PLGA NPs co-deliver or are paired with PD-L1 inhibitors and paclitaxel (PTX), leading to strengthened CD8^+^ T-cell responses through modulation of NF-κB and STAT1 signaling and programs related to epithelial–mesenchymal transition (EMT). Overall, PLGA NPs function as a modular platform for drug delivery and immune remodeling, integrating antigen exposure, APC activation, macrophage repolarization, and checkpoint blockade synergy to amplify antitumor immunity. PLGA, poly (lactic-co-glycolic acid); PTX, paclitaxel; PD-L1, programmed death-ligand 1; NF-κB, nuclear factor kappa B; STAT1, signal transducer and activator of transcription 1; EMT, epithelial–mesenchymal transition; APC, antigen-presenting cell; TLS, tertiary lymphoid structure.

Tumor-associated macrophages (TAMs) drive cancer progression by enforcing immune suppression within the TME (Geltz et al., 2025). Reprogramming TAMs represents an effective antitumor strategy by blocking M2-TAM–mediated cancer progression in CRC (Zhao et al., 2025). Peng and colleagues engineered a biomimetic PLGA-based nanoplatform that co-encapsulated artesunate and chloroquine for dual-targeted delivery. By cloaking the HPA drug-loaded core with a mannose-functionalized erythrocyte membrane (Man-EM), they generated HPA/AS/CQ@Man-EM, which suppressed colorectal cancer proliferation and reprograms tumor-associated macrophages, yielding robust antitumor efficacy in the preclinical models (Peng et al., 2023). Retinoic acid–loaded PLGA nanoparticles recalibrated STAT3 and NF-κB signaling pathways, mitigating immunosuppression and preventing M2-TAM–driven epithelial–mesenchymal transition (EMT) in CRC. Notably, preclinical studies suggested that combining these nanoparticles with anti–PD-L1 enhanced tumor control and delineated a promising therapeutic approach using anti–PD-L1–conjugated nanocarriers for aggressive CRC (Júnior et al., 2023). Despite widespread use, immunotherapy has not translated into substantial clinical benefit for many patients. A key contributor is the mildly acidic immunosuppressive tumor microenvironment. To boost activity within this niche, researchers have designed pH-responsive polymeric nanocarriers encapsulating interleukin-12 (IL-12). At tumor-relevant pH, these IL-12 platforms reprogrammed macrophages by diminishing M2-like markers and elevating proinflammatory M1-like markers (Silveira et al., 2025). Paclitaxel (PTX) is a cytotoxic chemotherapeutic used across many solid tumors, but its clinical utility is curtailed by acquired resistance, poor aqueous solubility, and dose-limiting toxicities such as peripheral neuropathy and myelosuppression (Marupudi et al., 2007). To mitigate these issues, a variety of nanotechnology-based delivery platforms have been engineered. Because chemotherapy alone often fosters resistance and tumor relapse, oligonucleotide-based immunotherapies are being investigated as monotherapies or in combination with cytotoxics. Among these, RNA interference has been widely explored, small interfering RNA (siRNA)-mediated post-transcriptional silencing of PD-L1 has shown anticancer promise (Chou et al., 2020). Therefore, by coupling paclitaxel-induced cytotoxicity with siRNA-driven suppression of tumor-overexpressed PD-L1, the combination therapy may curb proliferation and diminish cancer cell viability. Engineered nanoparticles consisting of a magneto–silica core sheathed with a pH-responsive PLGA layer, co-loaded with PTX and an siRNA targeting PD-L1, effectively suppressed PD-L1 expression and elicited apoptotic cell death. The combined action of PTX’s cytotoxicity and selective PD-L1 knockdown synergistically potentiated CD8^+^ T cell–dependent tumor cell cytolysis (Gupta et al., 2024). PLGA/gambogic acid (GA) nanoparticles were subsequently cloaked with membranes isolated from CRC cells to generate CCM–PLGA/GA NPs. This biomimetic nanovaccine employed GA as an immunoadjuvant while displaying neoantigens provided by the cell membrane. CCM–PLGA/GA NPs markedly promoted DCs maturation and fostered a pro-immunogenic tumor microenvironment conducive to antitumor immunity in CRC (Huang et al., 2023) (Figure 1).

Tertiary lymphoid structures (TLSs) are ectopic immune aggregates that arise within the TME (Li et al., 2025b). TLSs recapitulate key architectural and functional hallmarks of mature lymphoid organs, featuring coordinated organization of T- and B-cell zones, DC networks, and high endothelial venules that support lymphocyte trafficking, activation, and local adaptive immune responses (Zhao and Gao, 2025; Zhu, 2025). TLSs correlate with improved outcomes in CRC by converting the TME into an immune-active niche (Shen et al., 2025). Additionally, nanotechnology has shown significantly impacts on the regulation of TLSs and immune responses within CRC (Mi et al., 2025; Niu et al., 2024). For example, Hu et al. engineered a photoresponsive bacterial platform, E@L-P/ICG, in which microbes encapsulated the cytokine LIGHT and are cloaked with PLGA/indocyanine green (ICG) nanoparticles. After preferentially accumulating in orthotopic colonic tumors in mice, near-infrared laser exposure produced a mild photothermal rise via the P/ICG coating, triggering bacterial self-lysis and killing adjacent tumor cells. This process released bacterial adjuvants and tumor antigens that cooperatively primed adaptive immunity. In parallel, LIGHT liberated upon lysis driven the neogenesis of high endothelial venules (HEVs), enhancing lymphocyte recruitment within the TME and promoting *in situ* assembly of TLSs in CRC. The resulting robust T- and B-cell infiltration and activation suppressed tumor progression and prolong survival, highlighting E@L-P/ICG as a light-controllable strategy to induce TLSs for CRC therapy (Hu et al., 2025).

The liver is the predominant site of CRC dissemination (Zhang et al., 2022a). Tumor cells traverse into the perisinusoidal space (space of Disse) by adhering to liver sinusoidal endothelial cells (LSECs), while Tregs dampen DCs antigen presentation (Wang et al., 2023). Tumor-associated neutrophils expel chromatin to form neutrophil extracellular traps, sequestering CRC cells in the liver and ultimately enhancing their invasive and metastatic potential (Chen et al., 2022; Li et al., 2023b). Notably, chondroitin sulfate synthase 1 (CHSY1) was upregulated in both primary CRC and hepatic metastases, where it reprogramed succinate metabolism and activated the PI3K/AKT/HIF1A axis, driving CD8^+^ T-cell exhaustion and elevating PD-L1 expression to facilitate liver colonization (Sun et al., 2023). Pharmacologic inhibition of CHSY1 with artesunate reduced hepatic metastasis and potentiated the efficacy of anti–PD-1 therapy (Sun et al., 2023). Moreover, PLGA nanoparticles co-delivering artesunate and indocyanine green (ICG) attenuated liver metastasis and further improved responses to PD-1 blockade (Sun et al., 2023). Together, these findings delineate actionable mechanisms and highlight CHSY1 as a promising therapeutic target, offering specific, translational strategies for the treatment of CRC liver metastases. Embedding molecular and pharmacological investigations within a translational framework during the experimental stage facilitates the identification of novel clinical interventions and expands therapeutic prospects for CRC liver metastases (Table 1).

**TABLE 1:** PLGA-based nanoparticles in colorectal cancer immunotherapy.

Nanoparticle	Research type	Therapeutic intervention	Mechanisms	Reference
HPA/AS/CQ@Man-EM	Preclinical study	Artesunate and chloroquine	Suppressed colorectal cancer proliferation and reprograms tumor-associated macrophages, yielding robust antitumor efficacy	82
CHO-PLGA-RA	Preclinical study	Retinoic acid and cholestero	Recalibrated STAT3 and NF-κB signaling pathways, mitigating immunosuppression and preventing M2-TAM–driven epithelial–mesenchymal transition (EMT)	83
dual-Fe3O4-SiO2-PLGA-PDA-PTX-siRNA NPs	Preclinical study	PTX and PDA	Induced cytotoxic effect of PTX combined with targeted silencing of PD-L1, which in turn improved CD8^+^ T cell-mediated cancer cell death	86
CCM-PLGA/GA NPs	Preclinical study	Gambogic acid	Activated the maturation of dendritic cells (DCs) and the formation of a positive anti-tumor immune microenvironment	87
E@L-P/ICG	Preclinical study	Cytokine LIGHT	Triggered the death of surrounding tumor cells to release adjuvants and antigens, which in turn synergistically activated the adaptive immune responses	27
PLGA-loaded Artemisinin and ICG probe	Preclinical study	Artemisinin	Reduced liver metastasis and enhanced the effect of anti-PD1 in colorectal cancer	98

Summarizes representative PLGA-based nanoparticle systems for colorectal cancer immunotherapy. For each entry, we indicate the study type (preclinical *in vitro*/*in vivo* CRC), payloads/interventions, and key mechanisms—particularly immune modulation: TAM repolarization, DC maturation, antigen/adjuvant release, PD-1/PD-L1 regulation, and EMT suppression via STAT3/NF-κB. Multifunctional PLGA composites (e.g., Fe3O4/SiO2, ICG, PDA) are included when immunologic effects were shown. Because models, doses, and endpoints vary, the table supports qualitative comparison of design features rather than head-to-head efficacy. Abbreviations: PTX, paclitaxel; ICG, indocyanine green; PDA, polydopamine; TAM, tumor-associated macrophage; DC, dendritic cell; EMT, epithelial–mesenchymal transition.

## 5 Challenges in the application of PLGA in cancer therapy

In the biomedical field, PLGA is among the few biodegradable polymers approved by the U.S. Food and Drug Administration (FDA); however, its application continues to face numerous technical and translational challenges (Zhang et al., 2022b; Kim et al., 2022). During formulation and storage, hydrolytic breakdown of PLGA yields acidic oligomers (lactic and glycolic acids), which acidify the particle microenvironment, lower the local pH, and promote protein denaturation, aggregation, deamidation, and acylation (Ding and Zhu, 2018; Feltrin et al., 2022). Process-related stresses—solvent–water interfacial exposure, shear, sonication, and residual solvents could further perturb protein secondary structure. At the system level, unresolved nanotoxicology concerns (e.g., ROS generation, complement activation, off-target organ accumulation) and poor size uniformity (high polydispersity) erode experimental reproducibility and complicate regulatory approval (Lim et al., 2022). Translation is additionally hindered by scale-up complexity, aseptic manufacturing constraints (e.g., limited feasibility of 0.22 μm sterile filtration for larger particles), and stringent quality control requirements for batch-to-batch consistency (Lim et al., 2022).

PEGylation can prolong circulation and attenuate opsonization but adds regulatory and safety complexity. Although PLGA is generally recognized as safe, PEGylated and ligand-functionalized systems require exhaustive physicochemical profiling (size, polydispersity, zeta potential, PEG grafting density, payload), evidence of batch consistency and long-term stability, and comprehensive preclinical packages including cytotoxicity, immunogenicity, pharmacokinetics, acute and chronic toxicity. Accelerated blood clearance, as well as the fate of degradation products, must be closely monitored. Manufacturing scale-up must tightly control particle attributes; conventional sterilization can alter colloidal properties; and PEG coronas may hinder cellular uptake and deep-tumor penetration (Jani et al., 2023). The presence of a PEG corona may curtail adhesion and endocytosis while impeding diffusion through the extracellular matrix, thereby compromising cellular uptake and intratumoral penetration. Achieving an appropriate balance between prolonged circulation and tumor delivery requires careful adjustment of PEG chain length/density or deployment of stimuli-cleavable PEG (Tang et al., 2022b; Zaichik et al., 2020).

Multiple complementary strategies are under active investigation. Protein integrity can be preserved via zinc-ion complexation, inclusion of basic/antacid excipients (e.g., Mg(OH)2, CaCO3, histidine buffers), acylation inhibitors, lyoprotectants, and core–shell architectures that decouple the cargo from acidic domains. Modulating surface charge from cationic to neutral or slightly anionic—using PEG, poloxamers, zwitterionic or mucopenetrating coatings—reduces electrostatic interactions with airway mucus and enhances diffusivity. Active targeting can be achieved by decorating particles with ligands against lung or tumor–specific receptors (e.g., ICAM-1, integrins, folate, transferrin; antibodies, peptides, aptamers, glycans), thereby improving cellular engagement and lesion accumulation (Dimchevska et al., 2017). Finally, stimuli-responsive designs deploy contextual triggers—acidic pH (tumor/endosome), disease-associated enzymes (MMPs, elastase), redox gradients (elevated GSH), or ROS to enact spatiotemporally controlled release, raising therapeutic indices while reducing systemic exposure. Integrating these approaches with microfluidic production, Quality-by-Design, and real-time PAT can tighten size distributions and enhance manufacturability for clinical translation (Crintea et al., 2023).

## 6 Conclusions and perspectives

PLGA has emerged as a cornerstone polymer for nanomedicine owing to its biodegradability, biocompatibility, and suitability for delivering molecules with poor solubility and membrane permeability (Su et al., 2021). PLGA nanoparticles increase the probability of tumor exposure, a key advantage in oncology that can support reduced dosing frequency while maintaining or improving therapeutic effect (Nath et al., 2024). Emerging evidence highlights an important role for PLGA nanoparticles in cancer immunotherapy, particularly CRC. This review provides an integrated synthesis of drug-loaded, PLGA-based nanocarriers for CRC immunotherapy and outlines key determinants of release and therapeutic performance. Nevertheless, CRC-specific evidence remains limited, precluding rigorous, head-to-head comparisons of PLGA with other biodegradable platforms. Accordingly, future work should clarify how PLGA nanoparticles modulate the CRC TME and shape immunotherapy responses.

Despite their favorable attributes, important challenges persist. PEGylated carriers can still undergo rapid *in vivo* clearance—especially upon repeat dosing—due to accelerated blood clearance driven by anti-PEG antibody formation, which narrows the therapeutic window and may precipitate hypersensitivity reactions, complicating clinical use (Han et al., 2025). In parallel, large-scale manufacturing remains a barrier: tight control over particle size, drug loading, PEG grafting density, sterility, and batch-to-batch reproducibility is technically demanding (Muddineti and Omri, 2022). Collectively, these limitations underscore the need for continued optimization of nanoparticle architecture and process engineering. Looking ahead, harmonized *in vitro*/*in vivo* standards, GMP/GLP-compliant workflows, and sustainability assessments—favoring natural or semi-synthetic matrices where appropriate—will be critical. Mechanism-informed materials design (including tunable or cleavable stealth coatings), proactive monitoring of anti-PEG immunity with potential patient stratification, and integrated, quality-by-design process development can help balance circulation with intratumoral delivery and enhance manufacturability. With these advances, PLGA and PEGylated PLGA platforms are well positioned for safe, effective, and scalable translation in oncology and immunotherapy.
